# Nickel alendronate

**DOI:** 10.1107/S1600536812022532

**Published:** 2012-05-26

**Authors:** Małgorzata Sikorska, Maria Gazda, Jaroslaw Chojnacki

**Affiliations:** a Chemical Faculty, Gdansk University of Technology, Narutowicza 11/12, Gdansk PL-80233, Poland; b Faculty of Applied Physics and Mathematics, Gdansk University of Technology, Narutowicza 11/12, Gdansk PL-80233, Poland

## Abstract

The title compound {sys­tematic name: bis(μ_2_-dihydrogen 4-aza­niumyl-1-hy­droxy­butane-1,1-di­phos­pho­n­ato)bis­[aqua­(dihydrogen 4-aza­nium­yl-1-hy­droxy­butane-1,1-diphospho­n­ato)nickel(II)] dihydrate}, [Ni_2_(C_4_H_12_NO_7_P_2_)_4_(H_2_O)_2_]·2H_2_O, was synthesiized under hydro­thermal conditions. Its structure is isotypic with the Co^II^ analogue. The crystal structure is built up from centrosymmetric dinuclear complex mol­ecules and the structure is reinforced by a net of inter­molecular O—H⋯O and N—H⋯O hydrogen bonds. One water mol­ecule is bound to the Ni^II^ atom in the octahedral coordination sphere, while the second is part of the inter­molecular hydrogen-bond system.

## Related literature

For the isotypic Co^II^ compound, see: Man *et al.* (2006 ▶). For the structures and therapeutic properties of bis­phospho­nates, see: Russell (2011 ▶). For zinc alendronate, see: Dufau *et al.* (1995 ▶).
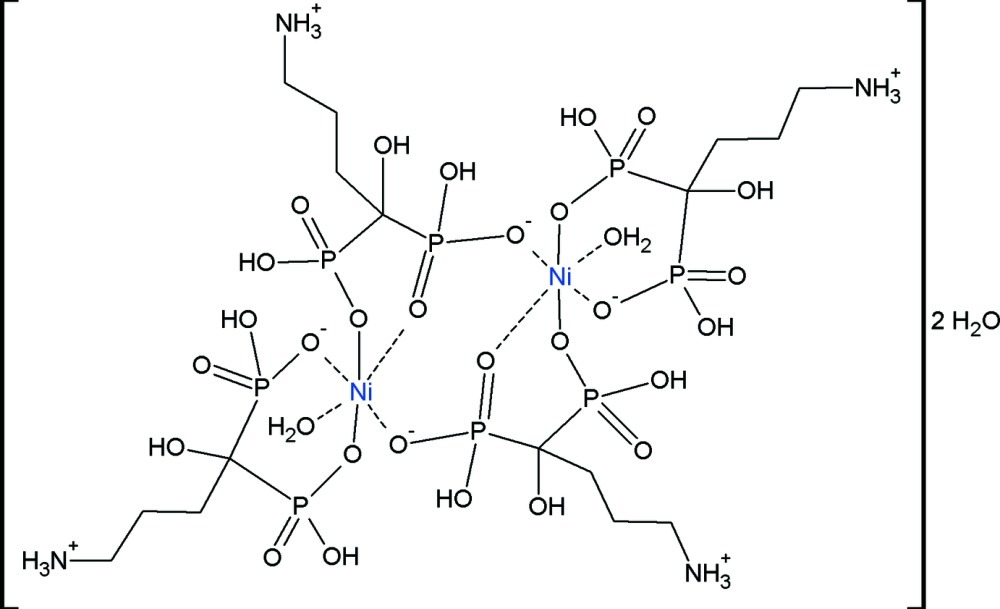


## Experimental

### 

#### Crystal data

[Ni_2_(C_4_H_12_NO_7_P_2_)_4_(H_2_O)_2_]·2H_2_O
*M* *_r_* = 1181.83
Monoclinic, 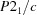

*a* = 12.5042 (3) Å
*b* = 13.5214 (2) Å
*c* = 12.4538 (3) Å
β = 109.667 (4)°
*V* = 1982.78 (9) Å^3^
*Z* = 2
Mo *K*α radiation
μ = 1.39 mm^−1^
*T* = 297 K
0.33 × 0.29 × 0.16 mm

#### Data collection

Oxford Diffraction KM-4-CCD Sapphire2 diffractometer
Absorption correction: analytical [*CrysAlis PRO* (Oxford Diffraction 2010 ▶), based on expressions derived by Clark & Reid (1995 ▶)] *T*_min_ = 0.747, *T*_max_ = 0.854
20727 measured reflections
3522 independent reflections
3237 reflections with *I* > 2σ(*I*)
*R*_int_ = 0.026

#### Refinement

*R*[*F* ^2^ > 2σ(*F* ^2^)] = 0.046
*wR*(*F* ^2^) = 0.124
*S* = 1.07
3522 reflections
298 parameters
6 restraints
H atoms treated by a mixture of independent and constrained refinement
Δρ_max_ = 2.44 e Å^−3^
Δρ_min_ = −0.67 e Å^−3^

### 

Data collection: *CrysAlis PRO* (Oxford Diffraction, 2010 ▶); cell refinement: *CrysAlis PRO*; data reduction: *CrysAlis PRO*; program(s) used to solve structure: *SHELXS97* (Sheldrick, 2008 ▶); program(s) used to refine structure: *SHELXL97* (Sheldrick, 2008 ▶); molecular graphics: *ORTEP-3* (Farrugia, 1997 ▶) and *Mercury* (Macrae *et al.*, 2008 ▶); software used to prepare material for publication: *WinGX* (Farrugia, 1999 ▶), Mercury, *publCIF* (Westrip, 2010 ▶) and *PLATON* (Spek, 2009 ▶).

## Supplementary Material

Crystal structure: contains datablock(s) global, I. DOI: 10.1107/S1600536812022532/zj2074sup1.cif

Structure factors: contains datablock(s) I. DOI: 10.1107/S1600536812022532/zj2074Isup2.hkl

Additional supplementary materials:  crystallographic information; 3D view; checkCIF report

## Figures and Tables

**Table 1:** Hydrogen-bond geometry (Å, °)

*D*—H⋯*A*	*D*—H	H⋯*A*	*D*⋯*A*	*D*—H⋯*A*
N1—H1*NA*⋯O11^i^	0.89	2.27	3.000 (4)	140
N1—H1*NB*⋯O4^ii^	0.89	2.13	2.980 (5)	159
N1—H1*NC*⋯O5^iii^	0.89	1.93	2.806 (4)	168
N2—H2*NA*⋯O2^iv^	0.89	2.43	3.215 (5)	147
N2—H2*NB*⋯O1	0.89	2.31	3.111 (5)	149
N2—H2*C*⋯O8^iv^	0.89	2.30	3.169 (6)	167
O2—H2⋯O5^ii^	0.82	1.68	2.487 (4)	170
O6—H6⋯O3^v^	0.82	1.73	2.539 (4)	168
O9—H9⋯O12^vi^	0.82	1.89	2.665 (4)	157
O11—H11⋯O8^iv^	0.82	1.78	2.585 (4)	165
O13—H13⋯O12^vi^	0.82	2.08	2.898 (4)	172
O15—H15*A*⋯O3^v^	0.83 (2)	2.00 (2)	2.815 (4)	168 (5)
O15—H15*B*⋯O16	0.82 (2)	2.29 (5)	2.882 (8)	130 (6)
O16—H16*A*⋯O8^vii^	0.88 (2)	2.57 (5)	3.365 (9)	151 (10)
O16—H16*B*⋯O8^iv^	0.88 (2)	2.03 (3)	2.902 (8)	169 (13)

Symmetry codes: (i) 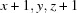; (ii) 
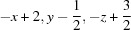
; (iii) 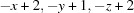; (iv) 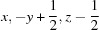; (v) 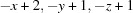; (vi) 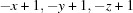; (vii) 
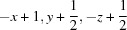
.

**Fig. 1.**
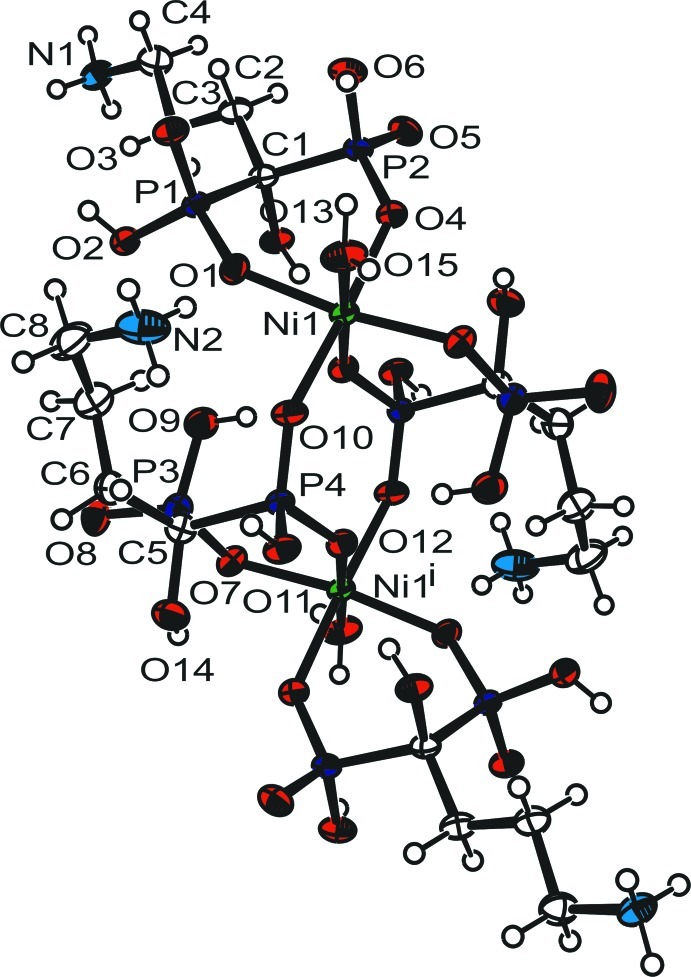
Molecular structure of C16H52N4Ni2O30P8 showing atom labeling scheme. Solvent water molecule not shown, displacement ellipsoids 50%.

**Fig. 2.**
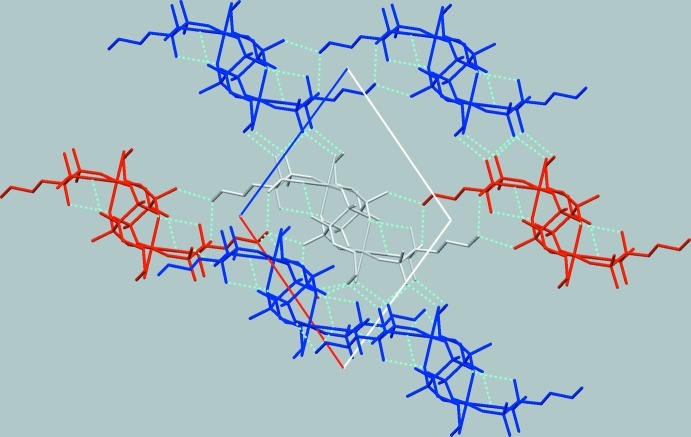
Packing diagram for C16H52N4Ni2O30P8 viewed along the b axis. Please note influence of different bending of the alkylammonium groups on hydrogen bonding system. Colours: central molecule - grey, molecules linked by N1—H···O5 bond - red, other neighbour molecules - blue

## supplementary crystallographic information

### Comment

Bisphosphonates are organic analogues of pyrophosphates with the P–O–P bridge replaced with a hydrolytically resistant P–C–P moiety. Their structure and therapeutic properties have been of vivid scientific interest for over 40 years (Russell, 2011). Bisphosphonates play essential role in modification of biomineralization in bones. Apart from the most important calcium salts, transition metal complexes are also being studied in respect to complex formation constants and X-ray structures *e.g.* to estimate and elucidate potential side effects of bisphosphonate drugs against osteoporosis. It was noticed (Man *et al.* 2006) that the length of side alkyl chain is crucial for determination of aggregation of metal bisphosphonates. For instance, in the case of Co bisphosphonates with six-carbon chain the mononuclear product was found, while the four-carbon hydrocarbon chain facilitated formation of the dinuclear complexes while shorter hydrocarbon side chains led to more or less complicated polymeric structures. Magnetic properties of the cobalt compounds have risen some interest and were examined in details.

Alendronic acid [CH(OH){(CH_2_)_3_NH_2_}{(PO(OH)_2_}_2_] in metal complexes usually occurrs as the zwitterionic monoanion with two P–OH groups deprotonated and the amino group protonated. Next two P–OH groups remain intact. Consequently divalent metals give neutral complexes (usually chelates) with metal to ligand ratio of 1:2.

The title compound was obtained from sodium salt of 4-amino-1-hydroxy-1,1-butylidenebisphosphonic acid and nickel(II) chloride in acidic aqueous solution. Both acidification and rising the temperature to *ca* 130 °C were necessary to obtain single crystals of X-ray quality. The product is insoluble in water and common organic solvents. The afforded crystals were investigated by single-crystal X-ray diffraction and additionally by microanalysis and powder diffraction in order to test the purity and composition of the whole batch.

The structure of the obtained compound, C_16_H_52_N_4_Ni_2_O_30_P_8_*2(H_2_O), turned out to be isomorphic with structure of cobalt derivative which was determined by Man *et al.* 2006. Structure composed of dinuclear complexes (though not isomorphic with the described above) was also found for zinc alendronate (Dufau *et al.* 1995). The text below recapitulates the main structural features of the determined structure.

Crystals are build up from centrosymmetric dinuclear complex molecules. Each metal atom coordination is close to octahedral, with one terminal water molecule, one terminal and two bridging bisphosphonate anions. All bisphosphonato ligands are chelating and contain one NH_3_^+^ and two —P(O)(O^-^)(OH) groups. The terminal ligands are bidentate, while the bridging ones are tridentate: one PO_3_H group is monodentate 1κ-O and the other is bridging bidentate 1κ-O',2κ-O'', using both nagatively charged O atoms and one oxygen atom from P=O group. Bond lengths allow only for general identification of P=O and P—O^-^ (*ca* 1.50 Å) or P–OH groups (*ca* 1.57 Å).

The system of hydrogen bonds is rather complex, see the relevant table. Packing of molecules is reinforced by O—H···O and by charge assisted (+)N—H···O hydrogen bonds. However, all internal hydrogen bonds can be easily recognized by the symmetry code of the acceptor atom being [-*x* + 1, -*y* + 1, -*z* + 1] (intramolecular inversion) or none. The alkylammonium chain N1 extends away from the core and forms only intermolecular hydrogen bonds with ligating and non ligating phosphonate O-atoms. Interesting *R*_2_^2^(16) centrosymmetric motif is formed by N1 ··· O5 bond around the *b* axis (see Figure 2.) The other alkylamonium chain is bent towards the central dinuclear core to facilitate intramolecular hydrogen bonding between the ammonium terminus and the O atoms. In fact, N2 ammonium groups form intramolecular as well as intermolecular hydrogen bonds. Hydroxyl group bound to carbon forms internal hydrogen bond O13—H ···O12[1 - *x*,1 - *y*,1 - *z*] and O14—H···O4[1 - *x*,1 - *y*,1 - *z*] and O14—H···O7. Water molecule (O15) bound to nickel atom forms hydrogen bonds with the next water molecule (O16) in the second coordination sphere. Apart from that extended intermolecular hydrogen bond network is present.

Microanalysis and powder diffraction pattern confirm the expected composition. Some discrepances between monocrystalic simulated intensities and experimental powder XRD intensities stem most likely from not uniform distribution of orientation of microcrystalites in the "powder" sample. Nevertheless, positions of all recorded peaks are correct.

### Experimental

Sodium alendronate (65 mg) was dissolved in 6 cm^3^ of water warmed to *ca* 70 °C. Then 4 ml of aqueous solution containing 95.2 mg of NiCl_2_.6H_2_O (0.4 mmole) and 0.5 ml of 2*M* HCl (1 mmole) were added. The pressure resistant container was closed and heated on an oil bath to 130 °C (inducing *ca* 3 bar overpressure) for 96 h. The content was let to cool slowly together with the oil bath and the obtained crystals were suitable for X-ray structural analysis. Elemental analysis (calculated for C_16_H_52_N_4_Ni_2_O_30_P_8_*2(H_2_O): C 16.34(16.26); H 4.71(4.77); N 4.75(4.74); S 0.0(0.0). Apparatus: Vario El Cube CHNS (Elementar), powder diffraction: *X*'Pert Philips diffractometer (Cu *K*_α_ radiation).

### Refinement

Structure was solved with all heavy atoms treated as anisotropic and H-atoms as isotropic. All C—H atoms were refined as riding on their bonded counterpart atoms with the usual constrains. Hydrogen atoms belonging to water molecules were refined with constrained O—H bond length to 0.84 Å. Two reflections (040) and (011) were identified as wrong and excluded from refinement.

### Crystal data

**Table:**

[Ni_2_(C_4_H_12_NO_7_P_2_)_4_(H_2_O)_2_]·2H_2_O	*F*(000) = 1224
*M**_r_* = 1181.83	*D*_x_ = 1.98 Mg m^−^^3^
Monoclinic, *P*2_1_/*c*	Mo *K*α radiation, λ = 0.71073 Å
Hall symbol: -P 2ybc	Cell parameters from 16141 reflections
*a* = 12.5042 (3) Å	θ = 2.3–28.8°
*b* = 13.5214 (2) Å	µ = 1.39 mm^−^^1^
*c* = 12.4538 (3) Å	*T* = 297 K
β = 109.667 (4)°	Block, green
*V* = 1982.78 (9) Å^3^	0.33 × 0.29 × 0.16 mm
*Z* = 2	

### Data collection

**Table:**

Oxford Diffraction KM-4-CCD Sapphire2 diffractometer	3522 independent reflections
Graphite monochromator	3237 reflections with *I* > 2σ(*I*)
Detector resolution: 8.1883 pixels mm^-1^	*R*_int_ = 0.026
ω scans	θ_max_ = 25.1°, θ_min_ = 2.3°
Absorption correction: analytical [*CrysAlis PRO* (Oxford Diffraction 2010), based on expressions derived by Clark & Reid (1995)]	*h* = −14→14
*T*_min_ = 0.747, *T*_max_ = 0.854	*k* = −16→16
20727 measured reflections	*l* = −14→14

### Refinement

**Table:**

Refinement on *F*^2^	Primary atom site location: structure-invariant direct methods
Least-squares matrix: full	Secondary atom site location: difference Fourier map
*R*[*F*^2^ > 2σ(*F*^2^)] = 0.046	Hydrogen site location: inferred from neighbouring sites
*wR*(*F*^2^) = 0.124	H atoms treated by a mixture of independent and constrained refinement
*S* = 1.07	*w* = 1/[σ^2^(*F*_o_^2^) + (0.0687*P*)^2^ + 6.2695*P*] where *P* = (*F*_o_^2^ + 2*F*_c_^2^)/3
3522 reflections	(Δ/σ)_max_ = 0.001
298 parameters	Δρ_max_ = 2.44 e Å^−^^3^
6 restraints	Δρ_min_ = −0.67 e Å^−^^3^

### Special details

**Table:**

Experimental. Absorption correction: CrysAlisPro, Oxford Diffraction 2010, Analytical numeric absorption correction using a multifaceted crystal model based on expressions derived by Clark & Reid 1995.
Geometry. All s.u.'s (except the s.u. in the dihedral angle between two l.s. planes) are estimated using the full covariance matrix. The cell s.u.'s are taken into account individually in the estimation of s.u.'s in distances, angles and torsion angles; correlations between s.u.'s in cell parameters are only used when they are defined by crystal symmetry. An approximate (isotropic) treatment of cell s.u.'s is used for estimating s.u.'s involving l.s. planes.
Refinement. Refinement of *F*^2^ against ALL reflections. The weighted *R*-factor *wR* and goodness of fit *S* are based on *F*^2^, conventional *R*-factors *R* are based on *F*, with *F* set to zero for negative *F*^2^. The threshold expression of *F*^2^ > 2σ(*F*^2^) is used only for calculating *R*-factors(gt) *etc*. and is not relevant to the choice of reflections for refinement. *R*-factors based on *F*^2^ are statistically about twice as large as those based on *F*, and *R*- factors based on ALL data will be even larger.

### Fractional atomic coordinates and isotropic or equivalent isotropic displacement parameters (Å^2^)

**Table:**

	*x*	*y*	*z*	*U*_iso_*/*U*_eq_	
Ni1	0.69424 (4)	0.54490 (3)	0.47489 (4)	0.01836 (16)	
P1	0.90380 (8)	0.39706 (7)	0.61692 (8)	0.0182 (2)	
P2	0.93083 (8)	0.61463 (7)	0.67849 (8)	0.0198 (2)	
P3	0.48228 (9)	0.27678 (8)	0.55326 (9)	0.0240 (2)	
P4	0.45576 (8)	0.41194 (7)	0.34777 (8)	0.0172 (2)	
N1	1.1817 (3)	0.2965 (3)	1.0790 (3)	0.0287 (8)	
H1NA	1.2515	0.2964	1.1300	0.043*	
H1NB	1.1698	0.2403	1.0396	0.043*	
H1NC	1.1313	0.3020	1.1149	0.043*	
N2	0.7320 (3)	0.3093 (4)	0.2984 (4)	0.0491 (11)	
H2NA	0.7813	0.3066	0.2610	0.074*	
H2NB	0.7470	0.3618	0.3441	0.074*	
H2C	0.6618	0.3140	0.2487	0.074*	
O1	0.7949 (2)	0.4259 (2)	0.5266 (2)	0.0250 (6)	
O2	0.8834 (2)	0.2960 (2)	0.6677 (2)	0.0246 (6)	
H2	0.9390	0.2603	0.6781	0.037*	
O3	1.0060 (2)	0.3913 (2)	0.5783 (2)	0.0254 (6)	
O4	0.8202 (2)	0.6342 (2)	0.5835 (2)	0.0240 (6)	
O5	0.9503 (2)	0.6825 (2)	0.7794 (2)	0.0277 (6)	
O6	1.0338 (2)	0.6234 (2)	0.6349 (2)	0.0270 (6)	
H6	1.0115	0.6177	0.5653	0.040*	
O7	0.3862 (2)	0.3254 (2)	0.5773 (2)	0.0240 (6)	
O8	0.5022 (3)	0.1702 (2)	0.5902 (3)	0.0340 (7)	
O9	0.5971 (2)	0.3324 (2)	0.6132 (2)	0.0301 (6)	
H9	0.5896	0.3911	0.5960	0.045*	
O10	0.5735 (2)	0.4502 (2)	0.3740 (2)	0.0244 (6)	
O11	0.3940 (2)	0.4038 (2)	0.2154 (2)	0.0249 (6)	
H11	0.4364	0.3770	0.1863	0.037*	
O12	0.3763 (2)	0.47229 (19)	0.3918 (2)	0.0212 (6)	
O13	0.8408 (2)	0.4841 (2)	0.7805 (2)	0.0244 (6)	
H13	0.7813	0.5021	0.7324	0.037*	
O14	0.3381 (2)	0.2459 (2)	0.3447 (3)	0.0320 (7)	
H14	0.3055	0.2448	0.3918	0.048*	
O15	0.7615 (3)	0.5602 (3)	0.3433 (3)	0.0350 (7)	
C1	0.9316 (3)	0.4880 (3)	0.7328 (3)	0.0203 (8)	
C2	1.0451 (3)	0.4727 (3)	0.8315 (3)	0.0257 (8)	
H2A	1.1049	0.4653	0.7987	0.031*	
H2B	1.0612	0.5324	0.8773	0.031*	
C3	1.0521 (3)	0.3859 (3)	0.9103 (4)	0.0299 (9)	
H3A	0.9952	0.3928	0.9469	0.036*	
H3B	1.0372	0.3249	0.8667	0.036*	
C4	1.1690 (4)	0.3818 (3)	0.9998 (3)	0.0304 (9)	
H4A	1.2255	0.3765	0.9624	0.037*	
H4B	1.1830	0.4428	1.0433	0.037*	
C5	0.4540 (3)	0.2832 (3)	0.3985 (3)	0.0243 (8)	
C6	0.5333 (3)	0.2156 (3)	0.3597 (3)	0.0271 (9)	
H6A	0.5072	0.1479	0.3587	0.033*	
H6B	0.5261	0.2331	0.2820	0.033*	
C7	0.6591 (4)	0.2194 (4)	0.4324 (4)	0.0369 (10)	
H7A	0.6723	0.2793	0.4779	0.044*	
H7B	0.6755	0.1638	0.4845	0.044*	
C8	0.7420 (4)	0.2174 (4)	0.3681 (5)	0.0463 (13)	
H8A	0.8187	0.2119	0.4217	0.056*	
H8B	0.7271	0.1601	0.3184	0.056*	
H15A	0.829 (2)	0.577 (5)	0.357 (5)	0.069*	
H15B	0.732 (4)	0.584 (5)	0.280 (3)	0.069*	
O16	0.6399 (6)	0.5061 (6)	0.1101 (7)	0.116 (2)	
H16A	0.584 (8)	0.532 (8)	0.054 (8)	0.175*	
H16B	0.605 (9)	0.448 (4)	0.102 (11)	0.175*	

### Atomic displacement parameters (Å^2^)

**Table:**

	*U*^11^	*U*^22^	*U*^33^	*U*^12^	*U*^13^	*U*^23^
Ni1	0.0144 (3)	0.0229 (3)	0.0179 (3)	0.00050 (18)	0.00562 (19)	−0.00056 (18)
P1	0.0149 (5)	0.0217 (5)	0.0171 (5)	0.0014 (4)	0.0041 (4)	−0.0012 (4)
P2	0.0161 (5)	0.0231 (5)	0.0200 (5)	−0.0026 (4)	0.0058 (4)	−0.0022 (4)
P3	0.0248 (5)	0.0255 (5)	0.0238 (5)	0.0026 (4)	0.0110 (4)	0.0032 (4)
P4	0.0159 (5)	0.0212 (5)	0.0151 (5)	0.0002 (4)	0.0059 (4)	−0.0006 (3)
N1	0.0223 (17)	0.039 (2)	0.0218 (17)	0.0053 (15)	0.0038 (14)	0.0006 (15)
N2	0.033 (2)	0.070 (3)	0.049 (3)	−0.013 (2)	0.0197 (19)	−0.004 (2)
O1	0.0211 (14)	0.0250 (14)	0.0234 (14)	0.0036 (11)	0.0003 (11)	−0.0042 (11)
O2	0.0201 (13)	0.0247 (14)	0.0289 (14)	0.0018 (11)	0.0079 (12)	0.0019 (11)
O3	0.0195 (13)	0.0352 (16)	0.0228 (14)	0.0004 (11)	0.0090 (11)	−0.0017 (12)
O4	0.0188 (13)	0.0249 (14)	0.0265 (14)	−0.0006 (11)	0.0052 (11)	0.0003 (11)
O5	0.0279 (14)	0.0288 (15)	0.0289 (15)	−0.0080 (12)	0.0128 (12)	−0.0084 (12)
O6	0.0199 (14)	0.0383 (17)	0.0236 (14)	−0.0073 (12)	0.0084 (11)	−0.0020 (13)
O7	0.0228 (14)	0.0291 (15)	0.0216 (13)	0.0060 (11)	0.0093 (11)	0.0047 (11)
O8	0.0457 (18)	0.0267 (16)	0.0394 (17)	0.0087 (13)	0.0271 (15)	0.0096 (13)
O9	0.0259 (15)	0.0345 (16)	0.0280 (15)	0.0012 (12)	0.0063 (12)	0.0035 (13)
O10	0.0162 (13)	0.0342 (16)	0.0228 (14)	−0.0026 (11)	0.0067 (11)	−0.0059 (11)
O11	0.0189 (13)	0.0356 (16)	0.0199 (13)	0.0009 (11)	0.0063 (11)	−0.0044 (11)
O12	0.0197 (13)	0.0259 (14)	0.0203 (13)	0.0014 (11)	0.0096 (11)	−0.0012 (11)
O13	0.0178 (13)	0.0358 (16)	0.0216 (13)	−0.0002 (12)	0.0093 (11)	0.0016 (12)
O14	0.0263 (15)	0.0354 (16)	0.0338 (16)	−0.0070 (13)	0.0092 (13)	−0.0078 (13)
O15	0.0235 (15)	0.056 (2)	0.0284 (16)	−0.0013 (14)	0.0128 (13)	0.0013 (14)
C1	0.0149 (17)	0.0248 (19)	0.0218 (18)	−0.0012 (15)	0.0070 (15)	−0.0015 (15)
C2	0.0184 (19)	0.035 (2)	0.0205 (19)	−0.0012 (16)	0.0025 (15)	−0.0009 (16)
C3	0.022 (2)	0.034 (2)	0.027 (2)	−0.0007 (17)	0.0001 (17)	0.0004 (17)
C4	0.027 (2)	0.038 (2)	0.0219 (19)	−0.0021 (18)	0.0030 (17)	0.0015 (17)
C5	0.024 (2)	0.025 (2)	0.0231 (19)	−0.0009 (16)	0.0079 (16)	0.0001 (16)
C6	0.029 (2)	0.027 (2)	0.027 (2)	0.0000 (17)	0.0113 (17)	−0.0069 (16)
C7	0.032 (2)	0.043 (3)	0.037 (2)	0.004 (2)	0.013 (2)	0.000 (2)
C8	0.029 (2)	0.056 (3)	0.052 (3)	0.011 (2)	0.012 (2)	−0.014 (3)
O16	0.117 (5)	0.110 (5)	0.128 (6)	−0.028 (4)	0.048 (4)	−0.009 (4)

### Geometric parameters (Å, º)

**Table:**

Ni1—O1	2.011 (3)	O7—Ni1^i^	2.017 (3)
Ni1—O7^i^	2.017 (3)	O9—H9	0.8200
Ni1—O10	2.054 (3)	O11—H11	0.8200
Ni1—O4	2.082 (3)	O12—Ni1^i^	2.139 (3)
Ni1—O15	2.089 (3)	O13—C1	1.449 (4)
Ni1—O12^i^	2.139 (3)	O13—H13	0.8200
P1—O1	1.497 (3)	O14—C5	1.466 (5)
P1—O3	1.511 (3)	O14—H14	0.8200
P1—O2	1.563 (3)	O15—O16	2.882 (8)
P1—C1	1.838 (4)	O15—H15A	0.83 (2)
P2—O5	1.508 (3)	O15—H15B	0.82 (2)
P2—O4	1.510 (3)	C1—C2	1.547 (5)
P2—O6	1.562 (3)	C2—C3	1.513 (6)
P2—C1	1.839 (4)	C2—H2A	0.9700
P3—O7	1.486 (3)	C2—H2B	0.9700
P3—O8	1.507 (3)	C3—C4	1.512 (5)
P3—O9	1.570 (3)	C3—H3A	0.9700
P3—C5	1.841 (4)	C3—H3B	0.9700
P4—O10	1.490 (3)	C4—H4A	0.9700
P4—O12	1.523 (3)	C4—H4B	0.9700
P4—O11	1.571 (3)	C5—C6	1.541 (5)
P4—C5	1.855 (4)	C6—C7	1.529 (6)
N1—C4	1.491 (6)	C6—H6A	0.9700
N1—H1NA	0.8900	C6—H6B	0.9700
N1—H1NB	0.8900	C7—C8	1.508 (6)
N1—H1NC	0.8900	C7—H7A	0.9700
N2—C8	1.496 (7)	C7—H7B	0.9700
N2—H2NA	0.8900	C8—H8A	0.9700
N2—H2NB	0.8900	C8—H8B	0.9700
N2—H2C	0.8900	O16—H16A	0.88 (2)
O2—H2	0.8200	O16—H16B	0.88 (2)
O6—H6	0.8200		
			
O1—Ni1—O7^i^	171.66 (11)	P4—O12—Ni1^i^	135.27 (16)
O1—Ni1—O10	87.07 (11)	C1—O13—H13	109.5
O7^i^—Ni1—O10	99.26 (11)	C5—O14—H14	109.5
O1—Ni1—O4	90.06 (11)	Ni1—O15—O16	123.4 (2)
O7^i^—Ni1—O4	83.75 (11)	Ni1—O15—H15A	121 (4)
O10—Ni1—O4	176.74 (11)	O16—O15—H15A	115 (4)
O1—Ni1—O15	87.52 (13)	Ni1—O15—H15B	129 (4)
O7^i^—Ni1—O15	87.12 (12)	H15A—O15—H15B	101 (3)
O10—Ni1—O15	89.36 (12)	O13—C1—C2	107.8 (3)
O4—Ni1—O15	92.05 (12)	O13—C1—P1	109.3 (2)
O1—Ni1—O12^i^	92.37 (11)	C2—C1—P1	114.5 (3)
O7^i^—Ni1—O12^i^	93.08 (10)	O13—C1—P2	106.1 (2)
O10—Ni1—O12^i^	89.82 (10)	C2—C1—P2	107.9 (3)
O4—Ni1—O12^i^	88.77 (10)	P1—C1—P2	110.97 (19)
O15—Ni1—O12^i^	179.18 (12)	C3—C2—C1	117.2 (3)
O1—P1—O3	115.32 (16)	C3—C2—H2A	108.0
O1—P1—O2	107.48 (16)	C1—C2—H2A	108.0
O3—P1—O2	110.75 (16)	C3—C2—H2B	108.0
O1—P1—C1	107.43 (16)	C1—C2—H2B	108.0
O3—P1—C1	109.11 (16)	H2A—C2—H2B	107.2
O2—P1—C1	106.33 (17)	C4—C3—C2	109.7 (3)
O5—P2—O4	113.25 (16)	C4—C3—H3A	109.7
O5—P2—O6	108.80 (16)	C2—C3—H3A	109.7
O4—P2—O6	111.05 (16)	C4—C3—H3B	109.7
O5—P2—C1	106.31 (17)	C2—C3—H3B	109.7
O4—P2—C1	109.97 (16)	H3A—C3—H3B	108.2
O6—P2—C1	107.18 (17)	N1—C4—C3	112.2 (4)
O7—P3—O8	115.04 (17)	N1—C4—H4A	109.2
O7—P3—O9	111.41 (17)	C3—C4—H4A	109.2
O8—P3—O9	106.36 (18)	N1—C4—H4B	109.2
O7—P3—C5	107.87 (17)	C3—C4—H4B	109.2
O8—P3—C5	108.56 (18)	H4A—C4—H4B	107.9
O9—P3—C5	107.33 (17)	O14—C5—C6	107.1 (3)
O10—P4—O12	116.83 (15)	O14—C5—P3	105.9 (3)
O10—P4—O11	110.80 (16)	C6—C5—P3	112.6 (3)
O12—P4—O11	105.41 (15)	O14—C5—P4	106.9 (3)
O10—P4—C5	112.03 (17)	C6—C5—P4	111.6 (3)
O12—P4—C5	107.24 (16)	P3—C5—P4	112.4 (2)
O11—P4—C5	103.50 (17)	C7—C6—C5	115.9 (3)
C4—N1—H1NA	109.5	C7—C6—H6A	108.3
C4—N1—H1NB	109.5	C5—C6—H6A	108.3
H1NA—N1—H1NB	109.5	C7—C6—H6B	108.3
C4—N1—H1NC	109.5	C5—C6—H6B	108.3
H1NA—N1—H1NC	109.5	H6A—C6—H6B	107.4
H1NB—N1—H1NC	109.5	C8—C7—C6	116.0 (4)
C8—N2—H2NA	109.5	C8—C7—H7A	108.3
C8—N2—H2NB	109.5	C6—C7—H7A	108.3
H2NA—N2—H2NB	109.5	C8—C7—H7B	108.3
C8—N2—H2C	109.5	C6—C7—H7B	108.3
H2NA—N2—H2C	109.5	H7A—C7—H7B	107.4
H2NB—N2—H2C	109.5	N2—C8—C7	110.8 (4)
P1—O1—Ni1	139.66 (17)	N2—C8—H8A	109.5
P1—O2—H2	109.5	C7—C8—H8A	109.5
P2—O4—Ni1	134.46 (17)	N2—C8—H8B	109.5
P2—O6—H6	109.5	C7—C8—H8B	109.5
P3—O7—Ni1^i^	131.97 (17)	H8A—C8—H8B	108.1
P3—O9—H9	109.5	O15—O16—H16A	135 (9)
P4—O10—Ni1	145.03 (17)	O15—O16—H16B	114 (8)
P4—O11—H11	109.5		
			
O3—P1—O1—Ni1	−101.7 (3)	O1—P1—C1—P2	−54.6 (2)
O2—P1—O1—Ni1	134.3 (3)	O3—P1—C1—P2	71.1 (2)
C1—P1—O1—Ni1	20.2 (3)	O5—P2—C1—O13	59.5 (3)
O10—Ni1—O1—P1	−167.7 (3)	O4—P2—C1—O13	−63.4 (3)
O4—Ni1—O1—P1	10.7 (3)	O6—P2—C1—O13	175.8 (2)
O15—Ni1—O1—P1	102.8 (3)	O5—P2—C1—C2	−55.7 (3)
O12^i^—Ni1—O1—P1	−78.0 (3)	O4—P2—C1—C2	−178.7 (2)
O5—P2—O4—Ni1	−138.7 (2)	O6—P2—C1—C2	60.5 (3)
O6—P2—O4—Ni1	98.6 (2)	O4—P2—C1—P1	55.1 (2)
C1—P2—O4—Ni1	−19.9 (3)	O6—P2—C1—P1	−65.7 (2)
O1—Ni1—O4—P2	−10.8 (2)	O13—C1—C2—C3	49.4 (5)
O7^i^—Ni1—O4—P2	174.8 (2)	P1—C1—C2—C3	−72.4 (4)
O15—Ni1—O4—P2	−98.3 (2)	P2—C1—C2—C3	163.5 (3)
O12^i^—Ni1—O4—P2	81.6 (2)	C1—C2—C3—C4	179.1 (4)
O8—P3—O7—Ni1^i^	−168.3 (2)	C2—C3—C4—N1	−179.1 (4)
O9—P3—O7—Ni1^i^	70.6 (3)	O7—P3—C5—O14	−50.5 (3)
C5—P3—O7—Ni1^i^	−47.0 (3)	O8—P3—C5—O14	74.7 (3)
O12—P4—O10—Ni1	16.8 (4)	O9—P3—C5—O14	−170.7 (2)
O11—P4—O10—Ni1	137.5 (3)	O7—P3—C5—C6	−167.2 (3)
C5—P4—O10—Ni1	−107.5 (3)	O8—P3—C5—C6	−41.9 (3)
O1—Ni1—O10—P4	121.6 (3)	O9—P3—C5—C6	72.6 (3)
O7^i^—Ni1—O10—P4	−63.8 (3)	O7—P3—C5—P4	65.8 (2)
O15—Ni1—O10—P4	−150.8 (3)	O8—P3—C5—P4	−168.9 (2)
O12^i^—Ni1—O10—P4	29.2 (3)	O9—P3—C5—P4	−54.3 (2)
O10—P4—O12—Ni1^i^	−106.8 (2)	O10—P4—C5—O14	−166.6 (2)
O11—P4—O12—Ni1^i^	129.6 (2)	O12—P4—C5—O14	63.9 (3)
C5—P4—O12—Ni1^i^	19.8 (3)	O11—P4—C5—O14	−47.2 (3)
O1—Ni1—O15—O16	104.2 (3)	O10—P4—C5—C6	−49.9 (3)
O7^i^—Ni1—O15—O16	−82.2 (3)	O12—P4—C5—C6	−179.3 (3)
O10—Ni1—O15—O16	17.1 (3)	O11—P4—C5—C6	69.5 (3)
O4—Ni1—O15—O16	−165.8 (3)	O10—P4—C5—P3	77.6 (2)
O1—P1—C1—O13	62.1 (3)	O12—P4—C5—P3	−51.8 (2)
O3—P1—C1—O13	−172.3 (2)	O14—C5—C6—C7	−161.5 (4)
O2—P1—C1—O13	−52.8 (3)	P3—C5—C6—C7	−45.5 (4)
O1—P1—C1—C2	−177.0 (3)	P4—C5—C6—C7	81.9 (4)
O3—P1—C1—C2	−51.3 (3)	C5—C6—C7—C8	−139.6 (4)
O2—P1—C1—C2	68.2 (3)	C6—C7—C8—N2	66.2 (5)

Symmetry code: (i) −*x*+1, −*y*+1, −*z*+1.

### Hydrogen-bond geometry (Å, º)

**Table:**

*D*—H···*A*	*D*—H	H···*A*	*D*···*A*	*D*—H···*A*
N1—H1*NA*···O11^ii^	0.89	2.27	3.000 (4)	140
N1—H1*NB*···O4^iii^	0.89	2.13	2.980 (5)	159
N1—H1*NC*···O5^iv^	0.89	1.93	2.806 (4)	168
N2—H2*NA*···O2^v^	0.89	2.43	3.215 (5)	147
N2—H2*NB*···O1	0.89	2.31	3.111 (5)	149
N2—H2*C*···O8^v^	0.89	2.30	3.169 (6)	167
O2—H2···O5^iii^	0.82	1.68	2.487 (4)	170
O6—H6···O3^vi^	0.82	1.73	2.539 (4)	168
O9—H9···O12^i^	0.82	1.89	2.665 (4)	157
O11—H11···O8^v^	0.82	1.78	2.585 (4)	165
O13—H13···O12^i^	0.82	2.08	2.898 (4)	172
O15—H15*A*···O3^vi^	0.83 (2)	2.00 (2)	2.815 (4)	168 (5)
O15—H15*B*···O16	0.82 (2)	2.29 (5)	2.882 (8)	130 (6)
O16—H16*A*···O8^vii^	0.88 (2)	2.57 (5)	3.365 (9)	151 (10)
O16—H16*B*···O8^v^	0.88 (2)	2.03 (3)	2.902 (8)	169 (13)

Symmetry codes: (i) −*x*+1, −*y*+1, −*z*+1; (ii) *x*+1, *y*, *z*+1; (iii) −*x*+2, *y*−1/2, −*z*+3/2; (iv) −*x*+2, −*y*+1, −*z*+2; (v) *x*, −*y*+1/2, *z*−1/2; (vi) −*x*+2, −*y*+1, −*z*+1; (vii) −*x*+1, *y*+1/2, −*z*+1/2.
